# Supplementary material for: Effectiveness of BMP-2 and PDGF-BB Adsorption onto a Collagen/Collagen-Magnesium-Hydroxyapatite Scaffold in Weight-Bearing and Non-Weight-Bearing Osteochondral Defect Bone Repair: In Vitro, Ex Vivo and In Vivo Evaluation
Source: J Funct Biomater. 2023 Feb 16;14(2):111. doi: 10.3390/jfb14020111 (PMC9961206; doi:10.3390/jfb14020111)
Supplement: Supplementary file 1 [file jfb-14-00111-s001.zip › Supplementary Material.pdf]

Supplementary Material

Table S1. Clinical Orthopedic Assessment. This tool is to evaluate the clinical health of the goat stifle joint.

Parameter	Variables	Score
Lameness	Walks normally	5
	Slightly lame when walking	4
	Moderately lame when walking	3
	Severely lame when walking	2
	Reluctant to rise and will not walk more than five paces	1
Joint mobility	Full range of motion	5
	Mild limitation (10–20%) in ROM; no crepitus	4
	Mild limitation (10–20%) in ROM; with crepitus	3
	Moderate limitation (20–50%) in ROM; ±crepitus	2
	Severe limitation (>50%) in ROM; ±crepitus	1
Pain on knee palpation and	None	5
movement	Mild signs; Caprine turns head in recognition	4
	Moderate signs; Caprine pulls limb away	3
	Severe signs; Caprine vocalises or becomes aggressive	2
	Caprine will not allow palpation	1
Weight-bearing	Equal on all limbs standing and walking	5
	Normal standing; favours affected limb when walking	4
	Partial weight-bearing standing and walking	3
	Part. weight-bearing standing; non-weight-bearing walk	2
	Non-weight-bearing standing and walking	1
Overall score of clinical	Not affected	5
condition	Mildly affected	4
	Moderately affected	3
	Severely affected	2
	Very severely affected	1
Total score		25

Table S2. Macroscopic joint Assessment. This tool is to evaluate the macroscopic normalization of goat stifle joints when the joints were opened.

Parameter	Variables	Score
Wound healing abnormal	Yes	0
	No	1
Swelling of joints area	Yes	0
	No	1

Effusion of the joints	Yes	0
	No	1
Patellar luxation	Yes	0
	No	1
Joint mobility abnormal / Contractures	Yes	0
	No	1
Adhesions of whole joint	Yes	0
	No	1
Erosions of whole joint	Yes	0
	No	1
Synovial fluid abnormal	Yes	0
	No	1
Synovial membrane abnormal	Yes	0
	No	1
lesion on the opposite cartilage surface	Yes	0
(trochlear groove vs patella)	No	1
lesion on the opposite cartilage surface	Yes	0
(medial femoral condyle vs meniscus/tibia plateau)	No	1
	Total	0-11

Table S3. International Cartilage Repair Society (ICRS) cartilage repair scoring system [1].

**Parameter**	**Variables**	**scores**
Degree of defect repair	In level with surrounding cartilage	4
	75% repair of defect depth	3
	50% repair of defect depth	2
	25% repair of defect depth	1
	0% repair of defect depth	0
Integration to border zone	Complete integration with surrounding cartilage	4
	Demarcating border < 1 mm	3
	¾ of graft integrated, ¼ with a notable border > 1 mm	2
	1/2 of graft integrated with surrounding cartilage,1/2 with a notable border > 1 mm	1
	From no contact to ¼ of graft integrated with surrounding cartilage	0
Macroscopic appearance	Intact smooth surface	4
	Fibrillated surface	3
	Small, scattered fissures or cracks	2
	Several, small or few but large fissures	1
	Total degeneration of grafted area	0

Overall	Grade I normal	12
	Grade II nearly normal	11-8
	Grade III abnormal	7-4
	Grade IV severely abnormal	3-1

Table S4. A semi-quantitative macroscopic scoring system developed by Goebel et al. [2]

**Parameter**	**Variables**	**scores**
Color of the repair tissue	Hyaline or white	4
	Predominantly white (>50%)	3
	Predominantly translucent (>50%)	2
	Translucent	1
	No repair tissue	0
Presence of blood vessels in the repair tissue	No	4
	Less than 25% of the repair tissue	3
	25-50% of the repair tissue	2
	50-75% of the repair tissue	1
	More than 75% of the repair tissue	0
Degeneration of adjacent articular cartilage	Normal	4
	Cracks and/or fibrillations in integration zone	3
	Diffuse osteoarthritic changes	2
	Extension of defect into the adjacent cartilage	1
	Subchondral bone damage	0
Surface of the repair tissue	Smooth, homogeneous	4
	Smooth, heterogeneous	3
	Fibrillated	2
	Incomplete new repair tissue (rough)	1
	No repair tissue	0
Percentage defect filling	80-100%	4
	60-80%	3
	40-60%	2
	20-40%	1
	0-20%	0
Total Scores		20

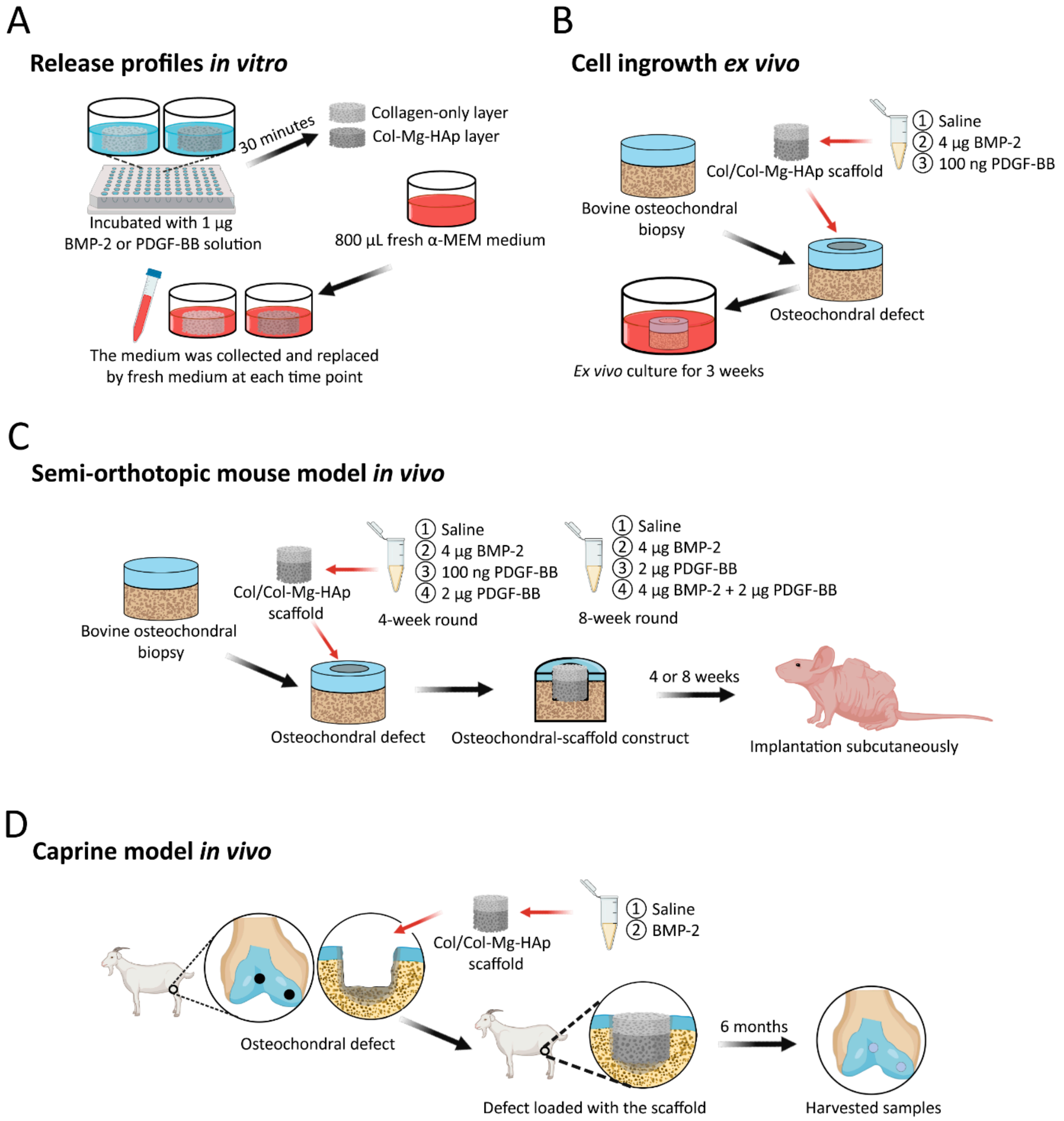

Figure S1. Experiment setup of both in vitro and in vivo studies. (A)Scheme of the in vitro release of BMP-2 and PDGF-BB from the different layers of the Col/Col-Mg-HAp scaffold. (B) Scheme of the ex vivo osteochondral defect culture model. (C) Scheme of the in vivo osteochondral defect mouse model. (D) Scheme of the in vivo osteochondral defect in medial femoral condyle and trochlear groove defects in caprine model.

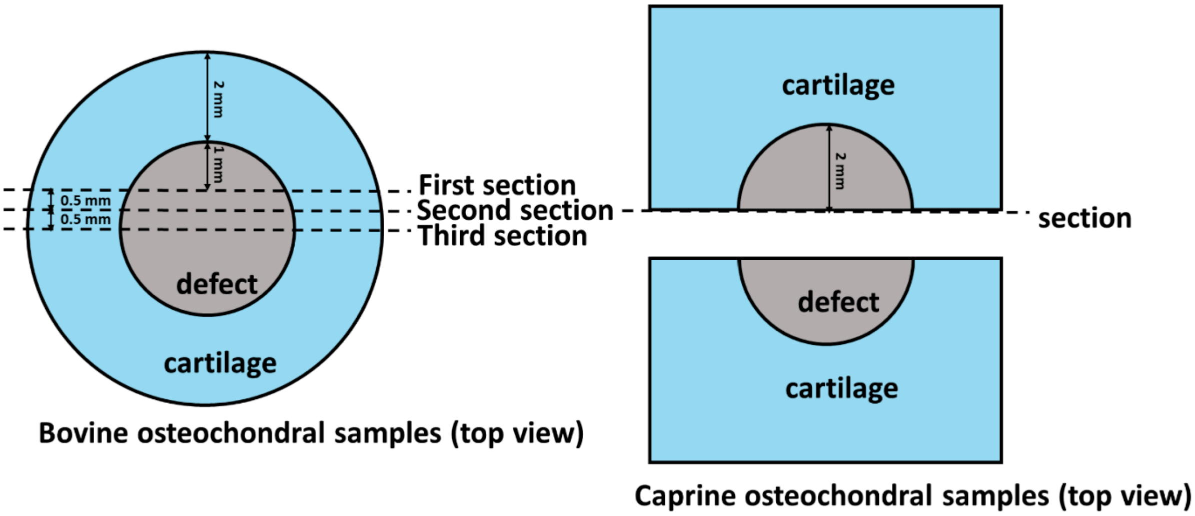

Figure S2. Collection of sections from bovine or caprine samples for histology.

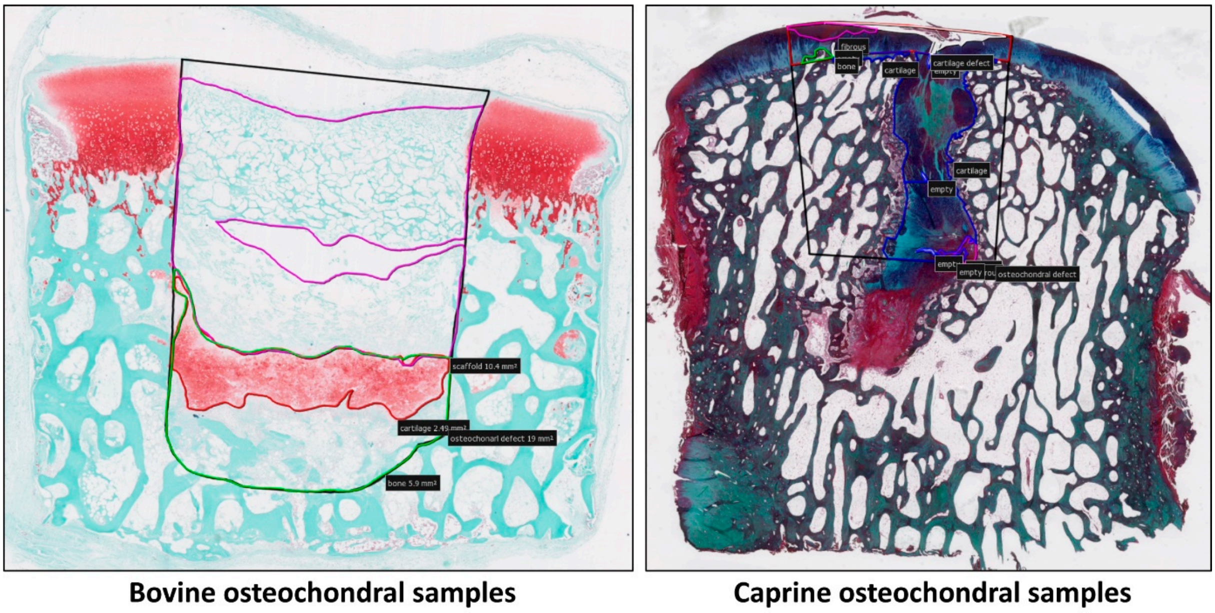

Figure S3. Example on defining the defect region, newly formed cartilage-like tissue formation, bone-like tissue formation, fibrous-like tissue formation, remnants of the scaffold for quantification.

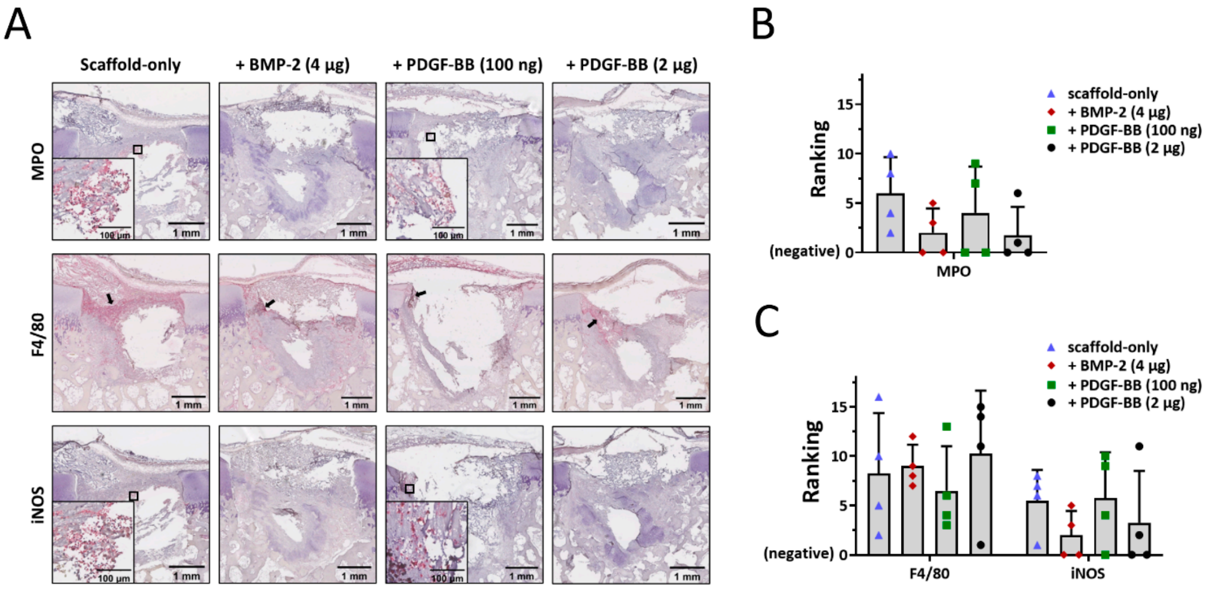

Figure S4. Association between inflammation and tissue repair at the early phase. (A) Representative images of the 4-week repair constructs stained with immunohistochemistry for MPO and iNOS. Scale bars indicate 1 mm and 100 µm respectively. Black arrows indicated positive areas. Ranking of MPO (B), F4/80 and iNOS (C) staining in the 4-week osteochondral defects.

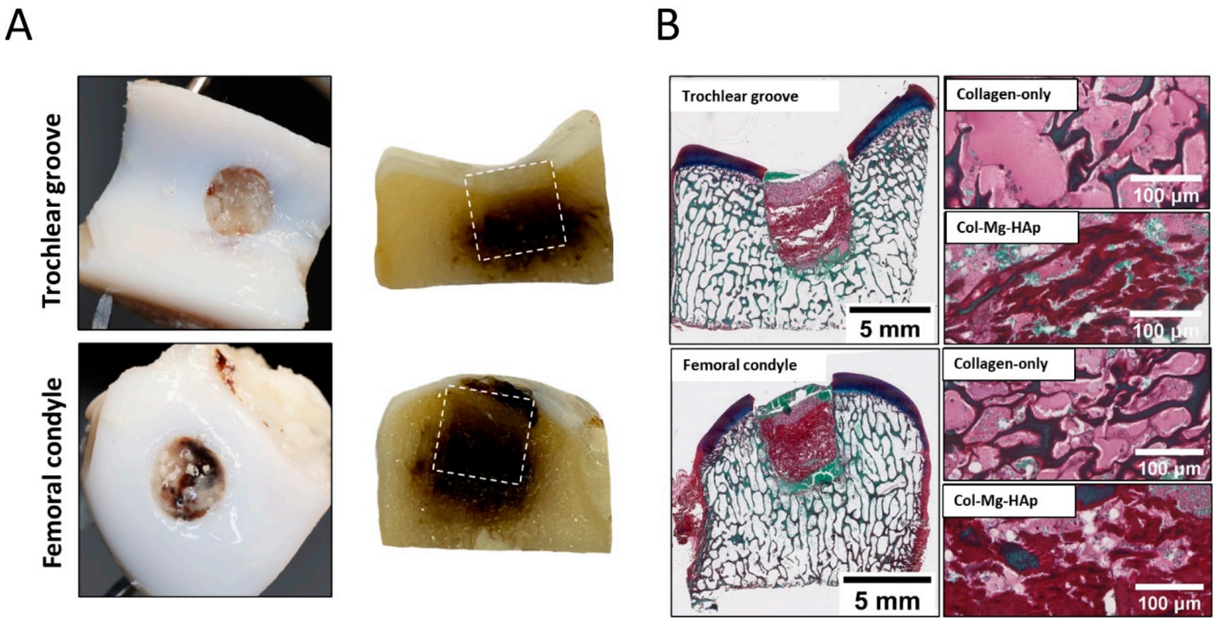

Figure S5. Osteochondral defects 3 days after implantation. (A) The macroscopic appearance of osteochondral defects 3 days after implantation. The white squares indicated 6*6 mm osteochondral defects. (B) two layers of the scaffold implanted in the femoral condyle defect and trochlear groove defect (stained with Alcian Blue, Fast Green, and Picrosirius Red). The scale bar indicated 5 mm and 100 µm.

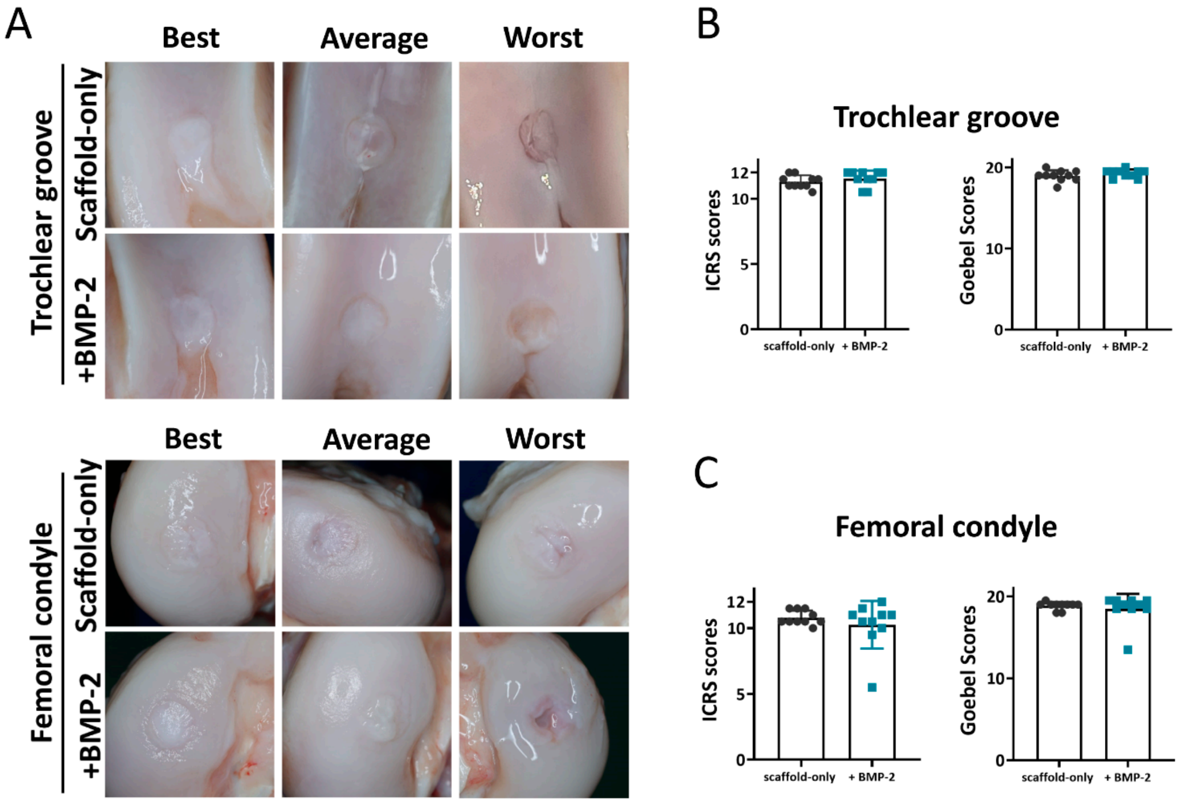

Figure S6. Macroscopic assessment of femoral condyle and trochlear groove defect repair. (A) representative examples of trochlear groove defect sites treated with scaffold-only or BMP-2-adsorbed scaffold after 6 months. Best, average, and worst samples determined according to the ICRS scores, are presented. (B) macroscopic scores (according to ICRS Score and Goebel Score) of repair tissue in the trochlear groove defects. (C) representative examples of femoral condyle defect sites treated with scaffold-only or BMP-2-adsorbed scaffold after 6 months. Best, average, and worst samples determined according to the ICRS scores, are presented. (D) macroscopic scores of repair tissue in the femoral condyle defects (according to ICRS score and Goebel Score). The maximum score for ICRS is 12 (indicating the best), and the maximum score for Goebel score is 20 (indicating the best).

References

1. van den Borne, M.P.; Raijmakers, N.J. International Cartilage Repair Society (ICRS) and Oswestry macroscopic cartilage evaluation scores validated for use in Autologous Chondrocyte Implantation (ACI) and microfracture. *Osteoarthr. Cartil.* 2007, 15, 1397-1402.
2. Goebel, L.; Orth, P. Experimental scoring systems for macroscopic articular cartilage repair correlate with the MOCART score assessed by a high-field MRI at 9.4 T--comparative evaluation of five macroscopic scoring systems in a large animal cartilage defect model. *Osteoarthr. Cartil.* 2012, 20, 1046-1055.
